# Mobile Phone Apps to Support Heart Failure Self-Care Management: Integrative Review

**DOI:** 10.2196/10057

**Published:** 2018-05-02

**Authors:** Ponrathi Athilingam, Bradlee Jenkins

**Affiliations:** ^1^ University of South Florida Tampa, FL United States

**Keywords:** heart failure, self-care management, mobile health

## Abstract

**Background:**

With an explosive growth in mobile health, an estimated 500 million patients are potentially using mHealth apps for supporting health and self-care of chronic diseases. Therefore, this review focused on mHealth apps for use among patients with heart failure.

**Objective:**

The aim of this integrative review was to identify and assess the functionalities of mHealth apps that provided usability and efficacy data and apps that are commercially available without supporting data, all of which are to support heart failure self-care management and thus impact heart failure outcomes.

**Methods:**

A search of published, peer-reviewed literature was conducted for studies of technology-based interventions that used mHealth apps specific for heart failure. The initial database search yielded 8597 citations. After filters for English language and heart failure, the final 487 abstracts was reviewed. After removing duplicates, a total of 18 articles that tested usability and efficacy of mobile apps for heart failure self-management were included for review. Google Play and Apple App Store were searched with specified criteria to identify mHealth apps for heart failure. A total of 26 commercially available apps specific for heart failure were identified and rated using the validated Mobile Application Rating Scale.

**Results:**

The review included studies with low-quality design and sample sizes ranging from 7 to 165 with a total sample size of 847 participants from all 18 studies. Nine studies assessed usability of the newly developed mobile health system. Six of the studies included are randomized controlled trials, and 4 studies are pilot randomized controlled trials with sample sizes of fewer than 40. There were inconsistencies in the self-care components tested, increasing bias. Thus, risk of bias was assessed using the Cochrane Collaboration’s tool for risk of selection, performance, detection, attrition, and reporting biases. Most studies included in this review are underpowered and had high risk of bias across all categories. Three studies failed to provide enough information to allow for a complete assessment of bias, and thus had unknown or unclear risk of bias. This review on the commercially available apps demonstrated many incomplete apps, many apps with bugs, and several apps with low quality.

**Conclusions:**

The heterogeneity of study design, sample size, intervention components, and outcomes measured precluded the performance of a systematic review or meta-analysis, thus introducing bias of this review. Although the heart failure–related outcomes reported in this review vary, they demonstrated trends toward making an impact and offer a potentially cost-effective solution with 24/7 access to symptom monitoring as a point of care solution, promoting patient engagement in their own home care.

## Introduction

### Background

Heart failure (HF) affects 6.5 million Americans and over 26 million people globally, which causes significant symptom burden and human suffering with considerable economic burden due to hospital readmissions [1,2]. The prevalence of HF is expected to increase 46% by 2030 [2]. A recent scientific statement from the American Heart Association indicates that self-care research and clinical efforts have been hindered by the perceptions of both patients and providers that pharmacological interventions are more effective than lifestyle change, thus warranting researchers to focus on self-management and lifestyle interventions for HF [3]. HF self-care requires patients to perform daily self-monitoring for changes in weight and symptoms, practice decision making for symptom changes, and adhere to prescribed medication, diet, physical activity, and follow-up care [4]. Living with HF imposes significant stressors for patients in following daily self-care tasks and lifestyle changes to maintain functional independence and quality of life [5]. Clinical outcomes in HF depend largely on how well a patient carries out self-care practices at home and seeks early care for symptoms. A metasynthesis of 47 studies recommends that self-care approaches must reflect both perception- and action-based strategies to effectively manage HF [5]. In order for self-care of any chronic condition such as HF to be sustained, self-management techniques need to be integrated into one’s lifestyle.

Given the complexity of HF self-care, assisting patients to manage their own care at home is a key component of HF management. Mobile health (mHealth) technology is defined as the “use of smartphones, tablets and other mobile devices to deliver health care and preventive health services” [6]. Given the explosive growth in mHealth for consumers, the World Health Organization predicted that over 500 million patients will be using mHealth apps by the end of this decade [6]. Personal mobile devices are portable and stay with the owner throughout the day. Thus, the mHealth market has taken root and seen exponential growth recently. Big technology companies such as Google, Apple, Microsoft, and IBM are partnering with health care organizations such as the American Diabetes Association and pharmaceutical companies in designing mHealth apps and systems to improve health care for people with chronic diseases [7]. Currently, mobile phones contain sensors such as accelerometers and cameras that have been leveraged in health care for health education, health management, and medical imaging such as electrocardiogram [8], as well as monitoring of pulmonary congestion in HF patients [9]. Among a sample of HF patients, 96% owned a mobile phone and 32% relied on the mobile phone for Internet access, searched health information, and reported moderate self-confidence in using mobile apps [10].

Emerging evidence suggests that mobile technology can serve as a form of support for patients with HF and may enhance patient-provider collaboration for self-management [11].

An integrative review of 11 studies provided an insight into user perception and experience with mobile apps: regardless of the user’s age and experience, mHealth tracking apps offered a sense of empowerment and control of chronic health conditions [12]. According to the Pew Research Center, 40% of American adults use mobile phones, which has more than doubled since 2013 [7]. Given the growing trend in the use of mHealth technology and mobile phone apps, this review is timely to discuss available evidence on the use of mHealth in HF self-care.

### Rationale for This Review

About 95% of Americans own a mobile phone of some kind and 40% of American adults are “mobile phone–only” Internet users [7]. Over 50% of mobile phone users are projected to have downloaded at least 1 health app to their phone in 2017 [7]. Currently, more than 150,000 apps are available, of which about 40,000 are mHealth apps for self-care management of chronic diseases commercially available on the market for management of asthma [13], diabetes [14], depression [15], smoking cessation [16], and weight management [17]. Over 700 apps are specifically available for patients with cardiovascular disease, many including blood pressure and heart rate tracking [18]. According to a review, 34 apps are commercially available for use by HF patients that have no established usability or efficacy data [19]. Other systematic reviews provided evidence on the use of telephone-based technology or structured telephone support [20] and remote telemonitoring [21-24]. Given the emerging evidence on the usability and efficacy of mHealth and the use of mobile phone apps, another review is deemed necessary to describe the current body of literature on mHealth apps for self-care management in HF. A prior review of HF apps included commercially available mobile apps [19], and a different review missed several newer mobile apps and systems [24]. Therefore, this review focused on mHealth apps and systems that included usability or potential efficacy tested on patients with HF and differentiated the commercially available mobile apps in the market.

### Aim of This Review

Mobile health is defined in detail by the World Health Organization as “the use and capitalization on a mobile phone’s core utility of voice and short messaging service as well as more complex functionalities and applications including general packet radio service, third and fourth generation mobile telecommunications (3G and 4G systems), Global Positioning System, and Bluetooth technology” [6]. Health apps are defined as any commercially available health or fitness apps with capacity for self-monitoring and improving patient compliance with treatment recommendations [6].

There has been explosive growth in mHealth apps with an estimated 500 million patients potentially using mHealth apps for supporting health and self-care of chronic diseases. However, commercially available apps are often not tested for usability and efficacy. The purpose of this review was to identify and assess the functionalities of mHealth apps that have usability and efficacy data and are available commercially on the market. All of the mHealth apps reviewed support HF self-care to improve treatment compliance, thus impacting HF outcomes.

The most effective self-management strategies include complex medication regimen, diet, and exercise recommendations and modification of behavior according to HF symptoms [25]. Protocol-driven disease management along with education, self-monitoring of HF symptoms, a flexible diuretic regimen, early care-seeking, prompt health care responses, psychosocial interventions, and professional coordination are successful strategies for self-management [25]. Less than half a percentage change on behavioral outcomes results from each self-care component, thus prompting recommendation of interventions with multiple self-care components for a cumulative effect on behavioral outcomes [26]. Also, novel mHealth technologies are recommended to serve as conduits for self-management in HF [27]. Therefore, given the complexity of HF self-management, multiple self-care components specific for patients with HF are recommended to achieve desired health outcomes [28]. A meta-analysis of 66 clinical trials from 18 countries recommended multiple strategies to reduce HF readmissions [29].

## Methods

### Objective

This review has 2 parts and is part of an overarching project on the development of a comprehensive mHealth app for HF self-management. The first part identifies mHealth apps that have been tested for usability and potential efficacy of mHealth apps for HF patients. The second part explored commercially available apps for HF that lacked usability and efficacy data, and apps were rated using the Mobile Application Rating Scale (MARS) [30].

### Selection Criteria

For the first part of the review, studies were included if they met the following criteria: (1) used a randomized controlled trial or quasi-experimental design or a pre-post-test design, (2) provided usability or potential efficacy data, (3) tested interventions using a mobile platform (by itself or as part of a mobile system), (4) included HF patients aged 18 years and older, and (5) were published in English. Excluded for part 1 were (1) studies that tested traditional remote telemonitoring interventions, transitional care, and structured telephone support in HF; (2) studies that tested mHealth apps on chronic conditions other than HF; (3) articles not in English; (4) studies that tested games; (5) studies that tested mHealth on other cardiac conditions such as cardiac rehabilitation and atrial fibrillation; (6) studies that included only clinical measures such electrocardiogram on mobile phones with no self-care measures; and (7) protocols and nonresearch articles. For part 2 of the review, commercially available apps for HF from Google Play and Apple App Store were included.

### Literature Search

A search of published, peer-reviewed literature was conducted for articles published from April 2008 to August 2017 that studied mobile technology-based interventions that used mobile apps specifically for HF. The researchers used key search terms to identify potential articles and systematic reviews and meta-analyses. Medline, PubMed, Cumulative Index to Nursing and Allied Health Literature, Embase, Web of Science, PsycINFO, Computer Source, Computers and Applied Sciences Complete, Journal of Medical Internet Research (JMIR), and Institute of Electrical and Electronics Engineers journal and conference proceedings were searched, and personal communications were included. Only articles published after 2008 were considered because that was the year the first app-ready mobile phone entered the market. For the second part of the review, Google Play and Apple App Store were searched for commercially available mHealth apps on the market for HF.

Keywords used: smartphone OR mobile phone OR mobile device OR tablet OR iPhone OR mobile technology OR iPad OR mHealth OR Android OR Windows; AND app OR apps OR mobile app OR application; AND heart disease OR heart failure; AND behavior OR behavior OR intervention OR controlled trial OR RCT.

### Search Result

**Figure 1:**
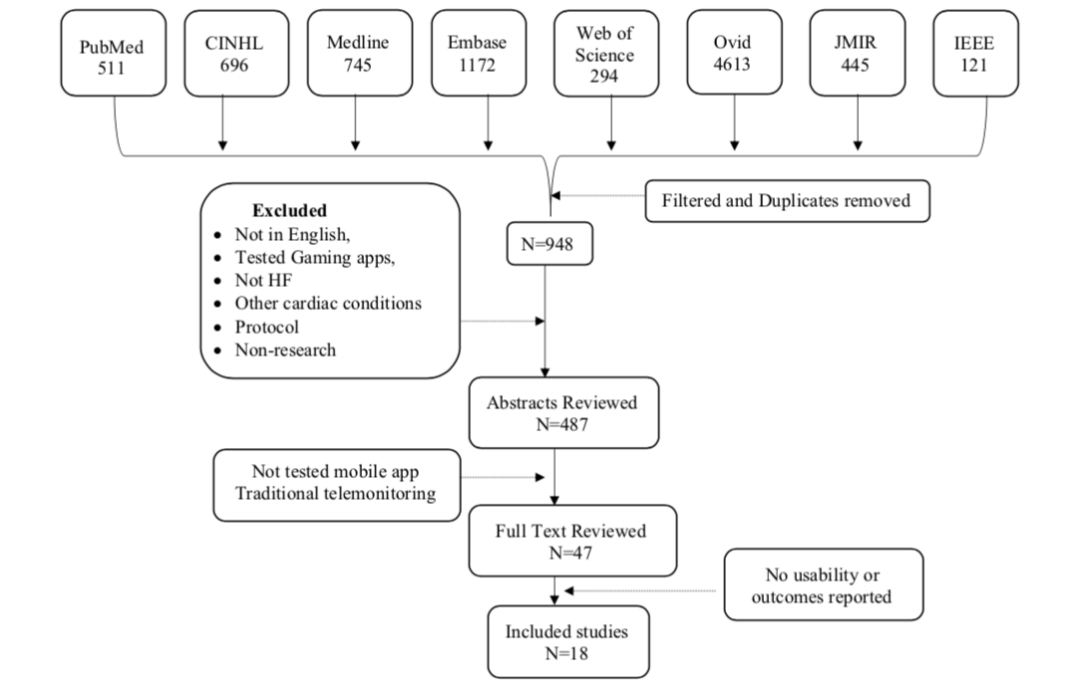
Flowchart on literature review and retrieval process.

The initial database search yielded 8597 citations from the 10 databases. After the predefined filters of language and HF were applied, 948 citations remained. A total of 487 abstracts remained after duplicates were removed. Each of the 487 abstracts was reviewed for articles that met the predetermined inclusion criteria. After articles that used traditional telemonitoring, remote telemonitoring, and other self-care interventions such as transitional care were excluded, 47 potential articles remained. Full-text evaluations of the 47 articles were conducted. Authors were contacted to obtain full-text articles if those were not available in PubMed or JMIR. Articles that did not test outcomes such as usability or potential efficacy and protocols were excluded. Finally, a total of 18 articles that tested mobile app or tablet-based mobile interventions in HF were included in this review (Figure 1). These mobile apps or systems are not available on the market.

For the second part, Google Play and Apple App Store were searched for health care apps with key words “heart failure,” “cardiac failure,” and “congestive heart failure.” The original search yielded over 4000 apps, which included apps for heart diseases. We excluded apps that were common for cardiovascular diseases, apps that track only blood pressure and heart rate, and apps that track general medications management or physical activity by syncing wearable such as the FitBit. After the exclusion criteria were applied, 56 mHealth apps from Google Play and Apple App Store were downloaded and examined to determine if they supported self-management in HF. Apps that included self-care components for general cardiovascular conditions such as hypertension or atrial fibrillation, tracked blood pressure, heart rate, or electrocardiogram for atrial fibrillation, or assessed heart rhythm were excluded. Finally, 26 apps that met the criteria for HF self-care were reviewed.

## Results

### Summary

In general, results indicated that mHealth apps for self-care in HF developed and tested recently are novel, and most are still testing usability in small numbers of HF participants. However, sample sizes from the 18 studies ranged from 7 to 165 with a total sample size of 847 participants from all 18 studies. The review was separated into apps that included efficacy outcomes data (Table 1) and apps that only included usability outcomes (Table 2).

All of the studies included in this first part of the review had usability or outcome assessment completed. Nine studies assessed usability of the newly developed mobile system, of which 7 included only usability data. Details of the studies included are depicted in Tables 1 and 2. Seven of the included studies were randomized controlled trials (RCTs), and 4 studies were pilot RCTs with sample sizes of less than 20 [30-33]. There were inconsistencies in the HF self-care components tested and included in the mobile app system, increasing bias, which is depicted in Multimedia Appendix 1. Risk of bias was assessed using the Cochrane Collaboration’s tool in all studies included. A few studies failed to provide enough information to allow for a complete assessment of bias (see Table 3).

After we applied the predetermined exclusion criteria, 56 mHealth apps from Google Play and Apple App Store were downloaded and examined for supporting self-management in HF. A total of 26 apps that met the criteria were rated using MARS [30] (see Table 4). MARS includes 19 items with 4 subscales that include engagement, functionality, aesthetics, and information quality [30]. The MARS items are scored using a 5-point Likert scale (1=inadequate, 2=poor, 3=acceptable, 4=good, and 5=excellent). MARS demonstrated excellent internal consistency (alpha=.90) and interrater reliability intraclass correlation coefficient (ICC=.79). MARS has been used by authors in evaluating commercially available mobile apps [19].

### Current Mobile Phone Interventions in Heart Failure

Ten of the 18 studies included in this review had small sample sizes of 40 or fewer participants (8 studies assessed usability of the mobile system for refinement or further development of an algorithm [31-40]), 4 studies had sample sizes of 41 to 99 [41-44], and 4 studies had 100 or more participants (but fewer than 200) [45-48]. Four of the 18 studies reviewed were pilot RCTs [32,34,42,44], and only 2 RCTs had 100 or more participants [47,48]. Total sample from all 18 studies was 847 participants. All 18 studies used mHealth technology via mobile phones or tablets as the medium for self-care intervention. A total of 11 studies reported outcome data (Table 1), 7 studies reported only usability of the mobile technology (Table 2), and 1 study developed an algorithm to differentiate HF but did not provide usability or efficacy data [40].

**Table 1:** Mobile health apps or systems that tested usability and/or potential efficacy on patients with heart failure.

Author	Study type	Intervention components	Outcomes measured	Results
Athilingam [32]	Pilot study (n=18) compared mobile app (HeartMapp) with HF education-only app, follow-up in 30 days	Symptom assessment, weight, blood pressure, medication management, alert and cues for action, mood and cognition assessment, deep breathing exercise for improved psychological health, track physical activity, support and communication with self-identified circle, track performance statistics	SCHFI^a^, KCCQ^b^, MMAQ^c^, AHFKT^d^, PHQ-9^e^, SE^f^, SUS^g^	Trends of improved self-management, knowledge, and QoL^h^. Usability established. Partial eta squared indicated small-to-moderate effect sizes (self-care 0.249, HF^i^ knowledge 0.337, QoL 0.156). Completion rate was 80%.
Austin [41]	Pre-post evaluation (n=60)	Daily interactive voice messages on educational tips	Readmission	Reduced readmission rate by 50% in 30 days; 25% dropout rate.
Dang [42]	RCT^j^ (n=61), intervention group (n=42), usual care (n=19)	Mobile system to alert for symptom assessment, weight, and blood pressure; mobile phone–assisted case management program in VA^k^ evaluated self-care efficacy, knowledge, behavior, and QoL; follow-up 3 months	EHFScBS^l^, DHFKS^m^, HDS^n^, HFSE^o^, MLHFQ^p^, SEMCD^q^, SF-36^r^	Usability established on minority population, program satisfaction scores (mean 6.84 [SD 0.46]), self-efficacy and QoL improved; 15% never used the app after enrollment.
Hagglund [44]	RCT (n=72), intervention (n=32), control group (n=40)	Weight and symptom assessment and HF education with a new system called home intervention system (HIS^s^, OPTILOGG)	EHFScBS, DHFKS, KCCQ, SF-36	Improved self-management, QoL, and physical function (all *P*<.05). Median adherence was 88%.
Hale [34]	Pilot RCT (n=25), intervention group (n=11), control (n=14)	MedCentry for medication management with alerts and dispensing medication on time. Measured medication adherence objectively on the device and subjectively using questionnaire	MLHFQ, MMAQ, PHQ-8^t^, readmission, usability of device	High medication adherence rate (95%), decreased hospitalization among intervention group 9% versus 50%, QoL improved (*P*=.02).
Pai [45]	Pre-post evaluation (n=130)	Weight, blood pressure, symptom assessment, alerts, medication management, track physical activity, used video education and clinician connect for care access	Readmission	53% reduction in readmission rate after rolling out the app.
Nundy [35]	Pilot, quasi-experimental (n=15)	Only text messages on HF education	SCHFI	Reported improved self-management (*P*=.002).
Piette [46]	Comparative effectiveness (n=165) study	Interactive voice response system with care partners in VA. Tracked weight, symptom assessment, medication management, alerts, and support system with care partners	NSSQ^u^, CES-D^v^	Improved medication adherence (*P*=.032).
Scherr [47]	RCT (n=108, equal 54 in groups)	MOBITEL platform used weight, blood pressure, and mobile phone for notification and access to data	Readmission, usability survey	Intention-to-treat 33% (1 death, 17 hospitalizations) in control group compared with 17% (0 deaths, 11 hospitalizations) in the intervention group; relative risk reduction 50% (95% CI 3%-74%, *P*=.06).
Seto [48]	RCT (n=100) mobile phone app and usual care with 6-month follow-up	Daily weight, symptom assessment, blood pressure, and EKG^w^ reading	SCHFI, MLHFQ, EKG, readmission	Improved self-care score (*P*=.03) and QoL (*P*=.05) among intervention group.
Suh [37]	Pilot study (n=26) pre-post evaluation	Weight, blood pressure, symptom assessment, reminder, and activity tracking	HFSAS^x^, readmission, observation of usability	Reported improved outcome on weight assessment. Patients reduced 5.6% of weight and blood pressure (*P*=.002).

^a^SCHFI: Self-Care of Heart Failure Index.

^b^KCCQ: Kansas City Cardiomyopathy Questionnaire.

^c^MMAQ: Morisky Medication Adherence Questionnaire.

^d^AHFKT: Atlanta Heart Failure Knowledge Test.

^e^PHQ-9: Patient Health Questionnaire–9 item.

^f^SE: Self-Efficacy Scale.

^g^SUS: System Usability Scale.

^h^QoL: quality of life.

^i^HF: heart failure.

^j^RCT: randomized controlled trial.

^k^VA: Veterans Administration.

^l^EHFScBS: European Heart Failure Self-Care Behavior Scale.

^m^DHFKS: Dutch Heart Failure Knowledge Scale.

^n^HDS: Health Distress Scale.

^o^HFSE: Heart Failure Self-Efficacy scale.

^p^MLHFQ: Minnesota Living With Heart Failure Questionnaire.

^q^SEMCD: Self-Efficacy for Managing Chronic Disease.

^r^SF-36: Short Form–36 item.

^s^HIS: home intervention system.

^t^PHQ-8: Patient Health Questionnaire–8 item.

^u^NSSQ: Norbeck Social Support Questionnaire.

^v^CES-D: Center for Epidemiologic Studies–Depression scale.

^w^EKG: electrocardiogram.

^x^HFSAS: Heart Failure Somatic Awareness Scale.

Despite the varying self-care components implemented, 8 of the 18 studies assessed HF-related readmission reporting a trend or significant reduction in readmission [31,34,37,38,41,45,47,48]. The Health Recovery Solution (HRS) Patient Connect study used a tablet-based mobile system that provided alerts; monitored weight, blood pressure, symptom assessment, and medication management; tracked physical activity; and used video education and clinician connect for care access. Postevaluation after implementing the intervention among 130 HF patients demonstrated a 50% decrease in HF-related readmission [45]. Implementing HF education alone demonstrated a significantly reduced 50% readmission rate in 30 days [49].

A comparative effectiveness study (n=165) in Veterans Administration (VA) patients demonstrated improved medication adherence [46]. Recently, a usability study among minorities in the VA population (n=61) reported moderate program satisfaction scores (mean 6.84 [SD 0.46]) and improved self-efficacy and quality of life [42]. Another comprehensive app established usability and improved self-management and quality of life in a pilot RCT [10,32]. Providing HF education via MP3 player showed reduced readmission rates [41]. Providing only mobile text messages on HF education in a pilot study improved self-management measured by a self-care of HF index questionnaire [35].

Seven of the included studies assessed self-management, of which 3 studies used the Self-Care of Heart Failure Index (SCHFI) [32,35,48], 2 studies used the European Heart Failure Self-Care Behavior Scale (EHFScBS) [42,44], 1 study used the Heart Failure Somatic Awareness Scale (HFSAS) [37], and 1 study used the personalized self-management system created within the mobile app [33]; all 7 of these studies reported improved self-management. Quality of life was measured by most studies, of which 5 used Minnesota Living With Heart Failure Questionnaire (MLHFQ) [31,34,39,42,48], 2 studies used the Kansas City Cardiomyopathy Questionnaire (KCCQ) [32,44], and 2 studies used the Short Form–36 (SF-36) [42,44]. All of the studies reported in general a trend for significant improvement in quality of life. The Norbeck Social Support Questionnaire (NSSQ) was used in 1 study to assess caregiver support [46]. The Dutch Heart Failure Knowledge Scale (DHFKS) was used to assess HF knowledge in 2 studies [42,44], and another used the Atlanta Heart Failure Knowledge Test (AHFKT) [32]. All 3 studies reported improved knowledge.

Two of the 18 studies measured depression using the Patient Health Questionnaire–9 (PHQ-9) [32,34], and another used the Center for Epidemiologic Studies–Depression (CES-D) scale [46]. Only 1 study used the data to offer deep breathing exercises to offset depression [32]. In addition to measuring self-care and quality of life, 1 study measured HF self-efficacy using the Self-Efficacy for Managing Chronic Disease (SEMCD) and program satisfaction scores and reported a moderate program satisfaction score (6.84 [SD 0.46]) [42]. Another study used the Short Portable Mental Status Questionnaire (SPMSQ) and the Technology Experience Questionnaire (TEQ) and reported overall adherence rates for blood pressure at 75%, weight at 82%, monitoring physical activity at 77%, and the mean usability rating among participants at 80% [43].

Some mHealth apps are in early stages of development in other countries: Canada [48], China [40], England [33], and Sweden [44]. One study measured N-terminal probrain natriuretic peptide (NT-proBNP) and other physiological measures of blood pressure and heart rate to develop a risk prediction model [40]. One study reported having an algorithm for fall detection with no result on fall or fall prevention [37]. In addition to self-care components, 1 study used single-lead electrocardiograms transmitting data to clinicians and reported inconclusive benefits; 14% of the patients randomized into the intervention group never used the system, and only 55% of the patients used the system at least 3 times per week [48].

Attrition or study completion was reported in 8 of the 18 studies, and adherence to mHealth intervention ranged from 50% to 80% [29,31,39-44]. Only the mobile system MedSentry, which not only reminded patients but also distributed medication on time, reported medication adherence, at 95% [34]. One study reported a 50% to 80% attrition rate because they gave every participant a locked phone with the mobile app, and patients reported that they did not like to carry an additional phone and wanted an app in their own phone [33]. Another study reported 80% completing a 30-day follow-up, and this decline in using the app was attributed to the chest-worn Bluetooth device used to track heart rate [32].

**Table 2:** Mobile health apps or systems that tested only usability on patients with heart failure.

Author	Study type	Interventions included	Outcomes measured	Results
Alnosayan [31]	Usability study (n=8) HF^a^, postdata collection at 6 months	Included symptom assessment, weight and blood pressure tracking, nurses followed patient report and supported patient	MLHFQ^b^, SUS^c^, readmission	User satisfaction was ranked at 73%.
Bartlett [33]	Usability assessed (n=7)	Symptom assessment, weight, and blood pressure, activity level, performance report, HF education. Patients were given a research phone, which they did not like to carry; wanted an app in their own phone	PSM^d^, SUS	Showed evidence of encouraging self-care, knowledge, and physical activity. Blood pressure was measured on 84% of the days, weight on 88%, walking for 51% of the days.
Evans [43]	Pilot usability study (n=41) among patients with HF and without HF using tablet	Weight and blood pressure, survey using tablet, track physical activity with a watch	SPMSQ^e^, SUS, TEQ^f^	Overall adherence for blood pressure 75%, weight 82%, watch monitor 77%. Usability rating was 80%. Adherence was reported 71% to 82%.
Portz [36]	Usability study (n=30), acceptability of the new HF symptom tracker app	Track daily weight and symptom assessment and give feedback as graph	Usability survey only	Usability established, mean score 3.5 (usability score ranged from 1.7 to 4.7). Older age was significantly associated with a self-identified need for help in the use of the HF app (r=.462, *P*=.01).
Triantafylidis [38]	Observation study (n=26), SUPPORT-HF^g^ (Seamless User-Centered Proactive Provision of Risk-Stratified Treatment for Heart Failure)	Used tablet computers and commercially available sensing devices (blood pressure monitor, set of weighing scales, and pulse oximeter), symptom-specific questionnaires, review their personal readings, view educational material	Readmission, observation for usability	Established usability; 23 patients (88%) used the system at least once and 16 patients (62%) used at least 3 times.
Zan [39]	Pilot feasibility study (n=21), follow-up 3 months	Web- and mobile-based intervention to monitor weight, blood pressure, heart rate, and symptoms	MLHFQ, SUS, satisfaction	Demonstrated feasibility; device under development based on feedback.
Zhang [40]	Pilot evaluation (n=34), 22 HF and 12 non-HF patients as controls and 30-day follow-up	Weight, blood pressure, physical activity, and HF symptom assessment. Offered feedback via text messages or emails from doctors.	HFRS^h^, HFSAS^i^, NT-proBNP^j^	SVM^k^-based mobile system that developed algorithm for HF risk prediction and determined prediction accuracy of 79.4%. No efficacy testing was done. The study is of poor quality.

^a^HF: heart failure.

^b^MLHFQ: Minnesota Living With Heart Failure Questionnaire.

^c^SUS: System Usability Scale.

^d^PSM: Personalized Self-Management System Score.

^e^SPMSQ: Short Portable Mental Status Questionnaire.

^f^TEQ: Technology Experience Questionnaire.

^g^SUPPORT-HF: Seamless User-Centered Proactive Provision of Risk-Stratified Treatment for Heart Failure.

^h^HFRS: Heart Failure Risk Score.

^i^HFSAS: Heart Failure Somatic Awareness Scale.

^j^NT-proBNP: N-terminal probrain natriuretic peptide.

^k^SVM: structured support vector machine.

**Table 3:** Assessment of risk of bias of the selected studies.

Studies	Selection bias	Performance bias	Detection bias	Attrition bias	Reporting bias
Alnosayan [31]	3^a^	3	2^b^	2	1^c^
Athilingam [32]	2	3	2	3	2
Austin [41]	3	3	2	3	2
Bartlett [33]	3	3	2	2	2
Dang [42]	2	2	3	2	2
Evans [43]	3	3	2	2	2
Hagglund [44]	2	2	2	3	2
Hale [34]	2	3	3	2	2
Pai [45]	2	1	1	1	1
Nundy [35]	3	2	2	2	2
Piette [46]	2	2	2	2	2
Portz [36]	2	3	1	2	2
Scherr [47]	2	3	2	2	2
Seto [48]	2	2	2	2	2
Suh [37]	3	3	2	2	2
Triantafylidis [38]	3	1	2	2	1
Zan [39]	3	3	3	2	2
Zhang [40]	3	2	3	3	2

^a^High risk of bias.

^b^Low risk of bias.

^c^Unknown risk of bias.

A total of 7 studies assessed only usability or patient satisfaction (see Table 2) in using the mobile app [31-33,36,38,39,42,43]; 2 studies that reported HF outcomes also assessed usability [32,42]. Two of the studies used survey methods to assess usability [36,47], and 2 studies observed patient data to measure usability of the components included in the mHealth app [37,38]. Four studies used validated questionnaires to measure usability of mHealth apps [31-33,43].

### Components of Heart Failure Self-Care Included in the Mobile Apps

All 18 studies included in this review varied widely on components of self-care management tested including medication management [32,34,46]. Most of the mobile technology included weight and symptom assessment [31-33,36-40,42-46,48], mobile messaging on HF self-management [32,35,40,41], and HF education [32,33,35,38,41,44,45]. Among the studies included, 7 of them considered mobile systems that included self-care components and clinical variable assessment. The HRS Patient Connect mobile system including weight, blood pressure, symptom assessment with feedback, medication management, and HF education using video reported reduced readmission rates at 3 months [45]. Similarly, 2 studies tested mobile systems among veterans and reported significantly improved HF outcomes [42,46]. Another mobile app, HeartMapp, tested acceptability and reported improved self-management in a pilot RCT [32]. HeartMapp is the only app that included biofeedback deep-breathing exercise to mitigate stress, 6-minute walk test to assess physical function, and mood and memory assessment [32]. In addition, 1 app included assessment of oxygen saturation [38], another included a single-lead electrocardiogram [48], and a third used NT-proBNP to develop an algorithm to calculate a risk score for HF, although this study did not include any usability or efficacy data [40] (see Multimedia Appendix 1).

Eight of 18 studies provide alerts or reminders to perform self-care. However, this aspect of the interventions is confusing, since data from these alerts are inconclusive or not reported, especially on the response to the alerts generated by the systems [31,32,34,37,41,42,45,46]. Only 1 study reported the reaction time (median 1 day, interquartile range 0 to 6) [35]. It is not known how many of these alerts were false or not responded to by participants. The other studies reported providing alerts and tracked their response with no results included.

### Assessment of Risk of Bias

The Cochrane Collaboration’s tool was used for assessing risk of bias in order to appraise the rigor of the included studies [50]. This tool has been tested in systematic reviews of health care interventions that frequently include RCTs [51] and non-RCTs [52]. This tool assesses for risk of selection, performance, detection, attrition, and reporting biases. Risk assessments of the included studies are presented in the form of a table containing the risk ratings (high, low, or unclear risk) [50]. In an effort to minimize various forms of research bias, studies are encouraged to assess for internal and external threats to validity. This tool was developed to assess risk of bias in RCTs and served as an objective measure to appraise bias of the HF studies included in this review. It should be mentioned that this analysis of the risks of bias is based on the current Cochrane tool; limitations and challenges still exist [53]. The Cochrane risk of bias tool aims to support researchers to enhance future study designs in order to translate them into practice.

The risk of bias tool was used because this tool is based on narrative descriptions of evidence-based methodological features known to increase the risk of bias in trials. Therefore, for a review that included studies with varying samples and pilot trials, we decided to use this tool to assess bias. The 2 authors of the study independently assessed the studies for bias. Most studies included in this review were underpowered and had high bias across all categories indicating varying ranges of methodological rigor. Studies that demonstrated high risk of selection bias provided inadequate data on randomization or lack thereof [31,33,35,37-41,43]. Several studies did not include information on blinding either the participants or study personnel, which increases internal threat to validity and results in high risk of performance bias [31-34,36,39,41,43,47]. There were only 2 studies that posed high risks of detection bias based on repeated testing methods [34,42]. Two studies have unknown risk of performance bias due to lack of information on blinding [38,45]. Attrition bias was determined if the outcome data were incomplete. Lastly, it was determined that all studies except the 3 found to lack information were determined to have unclear risks [31,38,45].

### Mobile Health Apps Commercially Available in the Market for Heart Failure

Forbes reported that over 50,000 mHealth apps are available that look to benefit our health, particularly physical fitness, mental health, general well-being, or management of chronic diseases [54]. As mentioned earlier, our search yielded 26 mHealth apps specific for HF self-care after applying our inclusion and exclusion criteria. These commercially available mobile apps have not been tested for usability or efficacy in impacting HF outcomes.

The 2 authors of this paper objectively evaluated and rated these commercial apps independently using MARS to determine the quality of the apps and if the apps support components of self-management of HF [30]. MARS functionality scores focus on performance, ease of use, navigation, and gestural design of the app. Functionality was assessed based on components of HF self-management, the type on self-management, amount of support or feedback provided, and quality of the app. Table 4 shows the 26 mHealth apps available on the market that are specific for use by HF patients with number of components and ratings included.

**Table 4:** Apps commercially available for patients with heart failure and number of self-care components monitored.

App name	Heart failure self-care components	Access/cost in USD	MARS^a^ score				
			Engagement	Functionality	Aesthetics	Information	Total MARS
AskMD patient app	7	Free	4.8	4.8	5.0	4.6	4.8
Heart Failure Health Storylines	7	Free	4.8	4.4	3.5	4.8	4.4
WebMD patient app	7	Free	4.0	4.6	4.5	4.2	4.3
Continuous Care Health App	6	Free	4.0	4.6	3.2	4.2	4.0
HeartKeeper	5	Free	4.0	4.2	3.4	4.4	4.0
Manage HF	6	$0.99	4.0	4.0	3.0	4.0	3.8
HF Defender	5	Free	3.8	4.0	3.0	3.8	3.7
WOW ME 2000mg	7	Free	3.0	4.6	3.0	3.0	3.4
Beat HF	4	Free	3.8	3.8	2.8	3.0	3.3
HeartPartner	5	Free	3.5	3.8	3.0	3.0	3.3
MyHeartApp	4	Free	3.0	3.2	2.8	3.0	3.0
Heart Failure coach	4	$49.99	3.5	3.0	3.0	3.0	3.1
Med-HF	4	Free	3.0	3.0	2.5	2.5	2.8
Health Manager	4	Free	3.0	3.0	2.0	3.0	2.8
HF Buddy	4	Free	3.0	3.0	2.0	3.2	2.8
Manage HF for Life	4	Free	2.8	3.0	2.0	2.5	2.6
MyHF	4	Free	2.5	3.0	2.0	2.0	2.4
Track your Heart Failure Zone	2	Free	2.0	1.0	1.0	1.0	1.3
HeartScrible	2	Free	1.0	1.0	1.0	1.0	1.0
Heart Log	2	Free	1.0	1.0	1.0	1.0	1.0
QardiyoHF	2	Free	1.0	1.0	1.0	1.0	1.0
My Symptom Guide	1	Free	1.0	1.0	1.0	1.0	1.0
Heart Failure monitoring	1	Free	1.0	1.0	1.0	1.0	1.0
SelfCare-MHR	1	Free	1.0	1.0	1.0	1.0	1.0
Signs and symptoms of HF	1	Free	1.0	1.0	1.0	1.0	1.0
Cardiio	1	Free	1.0	1.0	1.0	1.0	1.0

^a^MARS: Mobile Application Rating Scale.

## Discussion

### Principal Findings

In general, the first part of the review included studies that have tested an mHealth app in patients with HF. The second part included mHealth apps that are commercially available on the market for self-care of patients with HF. Most of the studies reviewed lack high-quality design and included a limited number of participants. Only 4 of the 18 studies reviewed included a sample size over 100 [45-48]. Of the 4, 2 were RCTs [47,48], 1 was a pre-post evaluation [45], and 1 was a comparative effectiveness analysis [46]. However, all 18 studies included 1 or more HF-specific self-care components provided on a mobile platform.

Most studies (14/18) monitored self-care components including weight, blood pressure, and HF symptoms. However, only 1 study assessed the severity of HF using a built-in algorithm based on the New York Heart Association classification. This classified patients into zones using American Heart Association categories of green, yellow, orange, and red zones and offered cues for action for values outside the range of the green zone [32]. Two studies reported using the HFSAS for HF symptom assessment with built-in algorithm but did not indicate if feedback was provided for patients to take action for values outside of range [37,40]. Most other apps only monitored the patients’ weight and HF symptoms and provided no feedback for values outside of range or left the responsibility to providers. This defeats the purpose of mHealth apps in helping patients develop a habit of adhering to self-care recommendations.

As indicated earlier, clinical outcomes in HF depend largely on how well a patient follows self-care and seeks care for symptoms early. Supporting patients through care transitions has been identified as a way to avoid hospital readmissions [55]. Mobile phones are carried by people and are always with them. Therefore, mHealth technology could potentially produce similar positive results if used beyond simple remote monitoring but targeted at behavior change based on behavior change theories, assisting patients to develop self-care as a habit and thus impacting outcomes. In a prior systematic review of 20 studies, the authors concluded that the use of technologies in managing HF patients at home had a positive impact on hospital readmission by 45%, improved mortality by 40%, and HF outcomes by 35% [56]. The key difference is that this review mainly focused on mHealth apps or systems that included 1 or more self-care components. Several of these studies demonstrated usability and potential efficacy for improving HF outcomes. Evidence shows that few mHealth apps are under development in countries such as Russia [57] and Iran [58]. Given the global public health burden of HF, large-scale studies to test these mHealth apps or systems for efficacy is warranted in America and around the world.

Attrition or study completion was reported in only half of the studies, with 1 study reporting as high as 80% attrition. In the study, researchers gave each participant a locked phone with the mobile app, and patients reported that they did not like to carry an additional phone and wanted an app on their own phone [33]. Only 1 study reported using participants’ own mobile phone to download the app [32], and other studies provided no details. A review recommended using a “bring your own device (BYOD)” strategy for research for sustainability [59]. The majority of people use their own mobile device for a variety of work-related communications and surfing the Internet. The BYOD strategy was successfully incorporated in education sectors [60] and thus is recommended for research to enhance customer satisfaction and long-term benefit [59].

Usability of the mHealth system was assessed in 9 studies, of which only 2 pilot studies reported potential efficacy along with usability [32,42] and 1 study provided only the algorithm developed for assessing risk [40]. Only 4 studies used validated questionnaires. None of the studies used the Android guidelines by Google that measure users’ quality of experience. A study that measured users’ quality of experience following Android guidelines by Google reported that many of the commercially available apps lack assessment of quality and usability, and many apps are low quality or incomplete with bugs [61]. The authors strongly recommend mHealth app developers use the recommended techniques to test quality of the apps before releasing them to market for customer use [61]. None of the 18 studies used the Android guidelines to assess their mHealth apps; 1 of the 26 apps available on the market was recently evaluated using Android guidelines and is undergoing refinement to fix flaws [62].

The prevalence rate of depression in HF patients ranges from 24% to 42%, and depression is graded as an independent risk factor for readmission to the hospital, functional decline, and mortality in patients with HF [63]. Three of the 18 studies measured depression or mood using the PHQ-9 or CES-D [32,42,46]. One study provided detail on mood assessment and offering deep-breathing exercise to offset depression [32]. Researchers need to keep this factor in mind as depression could reduce the use of the app. Various measures of physical function have been shown to predict rehospitalization and survival in patients with HF. Patients with HF commonly experience a highly variable symptom burden that is associated with reduced physical activity [64]. Six out of 18 studies tracked physical activity, and only 1 study included the 6-minute walk test to track progress and offer feedback [32]. The 6-minute walk test is a simple and useful prognostic marker for patients with mild-to-moderate HF [62]. Only 1 study reported tracking distance walked in 6 minutes to offer feedback on physical ability [34].

### Limitations

A major limitation of this review pertains to the characteristics of study design or methodology and a total sample of 847 from 18 studies that ranged from 7 to 165 participants. Most of the studies included are poor quality, with 4 studies having 100 or more participants, of which only 2 are RCTs, indicating a methodological bias. The other RCTs are pilot studies with small samples. A study with a sample as low as 7 subjects compromised generalizability, applications to practice, and utility of findings from this review. Finally, the heterogeneity of study design, intervention components, and outcomes measured precluded the performance of a systematic review, and thus introduced bias to this review. The review was solely conducted by the 2 authors of the review, and thus we feel that we may have included bias and the results are not generalizable. There could be other studies and other mHealth apps that this review may have missed.

This review included studies that tested mHealth apps for usability and potential efficacy in improving HF outcomes. The review also rated commercially available mHealth apps specific for HF that lacked evidence of usability or efficacy data. Although the HF-related outcomes reported in this review vary widely, a trend toward making an impact was observed. Mobile health may offer a potentially cost-effective solution with 24/7 access to symptom monitoring as a point of care solution in promoting patient engagement in their own care at home. Considering the novelty of mHealth interventions in HF and emerging evidence on mHealth app development around the world, we feel strongly that another review may be warranted.

### Future Research

Given the number of incomplete and poor-quality apps available on the market, focusing on improving apps that are already commercially available is a viable option. In addition, this review indicated that several mHealth apps for HF are under development around the world. mHealth app developers and researchers should collaborate with health care organizations and providers to align guideline-specific components in the app to improve outcomes. App developers are strongly advised to use the Android guidelines by Google to assess quality of user experience [61]. The authors recommend that the use of these tools by app developers may improve global acceptance of the apps and evaluation by the users. Evaluating the apps using valid tools such as MARS, complying with Google guidelines, and testing the apps for usability and potential efficacy of behavior change are warranted prior to making apps available for download by patients. The portability, connectivity, and touchscreen capabilities of mobile devices have the potential to revolutionize mHealth. As one author pointed out, “data integration should take place within the context of robust organizational governance frameworks that take into consideration the evaluation of clinical outcomes” [65]. App developers also should explore options for data migration within patient portals of electronic health records. In order for self-care of any chronic condition like HF to be sustained, self-management techniques need to be integrated into the patient’s life. Therefore, the researchers should also consider using a patient-centered approach during development of the app.

### Conclusion

This review indicates that mHealth in HF is novel, and new apps are under development. A few apps have assessed usability and are under development based on feedback from participants. The impact of mobile phone–based HF interventions on HF-related outcomes was inconclusive; however, use may enhance patient engagement in their care at home.

## Abbreviations

AHFKT: Atlanta Heart Failure Knowledge
BYOD: Bring Your Own Device
CES-D: Center for Epidemiologic Studies–Depression scale
DHFKS: Dutch Heart Failure Knowledge Scale
EHFScBS: European Heart Failure Self-Care Behavior Scale
EKG: electrocardiogram
HDS: Health Distress Scale
HF: heart failure
HFRS: Heart Failure Risk Score
HFSAS: Heart Failure Somatic Awareness Scale
HFSE: Heart Failure Self-Efficacy scale
HIS: home intervention system
HRS: Health Recovery System
ICC: intraclass correlation coefficient
KCCQ: Kansas City Cardiomyopathy Questionnaire
MARS: Mobile Application Rating Scale
MLHFQ: Minnesota Living With Heart Failure Questionnaire
MMAQ: Morisky Medication Adherence Questionnaire
NSSQ: Norbeck Social Support Questionnaire
NT-proBNP: N-terminal probrain natriuretic peptide
PHQ-8: Patient Health Questionnaire–8 item
PHQ-9: Patient Health Questionnare–9 item
PSM: Personalized Self-Management system score
QoL: quality of life
RCT: randomized controlled trial
SCHFI: Self-Care of Heart Failure Index
SE: Self-Efficacy scale
SEMCD: Self-Efficacy for Managing Chronic Disease
SF-36: Short Form–36 item
SPMSQ: Short Portable Mental Status Questionnaire
SUPPORT-HF: Seamless User-Centered Proactive Provision of Risk-Stratified Treatment for Heart Failure
SUS: System Usability Scale
SVM: structured support vector machine
TEQ: Technology Experience Questionnaire
VA: Veterans Administration

## Appendix

Multimedia Appendix 1 Heart Failure Self-Management Components Included in Studies Reviewed.
