# Supplementary material for: Mobile Phone Apps to Support Heart Failure Self-Care Management: Integrative Review
Source: JMIR Cardio. 2018 May 2;2(1):e10057. doi: 10.2196/10057 (PMC6834210; doi:10.2196/10057)
Supplement: Multimedia Appendix 1 [file cardio_v2i1e10057_app1.pdf]

	Heart Failure Self-Management Components Included																
	Alert	wt	BP	Symp	Med	Act	Edu	Mood	Supp	MM	Sync	Chart	BG	6MWT	DB	EKG	BNP
Alnosayan (2017) [31]	X	X	X	X					X			X	X				
Athilingam (2017)[32]	X	X	X	X	X	X	X	X	X	X	X	X		X	X		
Austin (2012)[41]	X						X			X							
Bartlett (2014)[33]		X	X	X													
Dang (2017)[40]	X	X	X	X				X	X								
Evans (2016)[43]		X	X			X	X				X						
Hagglund (2015)[44]		X	X			X	X										
Hale (2016)[34]	X				X												
Pai (2015)[45]	X	X		X	X	X	X				X						
Nundy (2013)[35]							X			X							
Piette (2015)[46]	X	X		X	X			X	X	X	X						
Portz (2017)[36]		X		X													
Scherr (2009)[47]		X	X		X				X								
Seto (2012)[48]		X	X	X								X				X	
Suh (2011)[37]	X	X	X	X		X			X		X	X					
Triantafylidis (2015)[38]		X	X	X			X		X			X					
Zan (2015)[39]		X	X	X								X					
Zhang (2017)[40]		X	X	X		X				X		X					X

Keys: Wt: weight; BP: Blood Pressure; Symp: Symptom Assessment; Med: Medication Management; Act: Physical Activity Tracking; Edu: Heart failure Education; Supp: Support communication with family and Providers; MM: Multimedia Message; Sync: Use other Bluetooth device to Sync with Mobile app; Chart: Performance Statistics as Graph; BG: Blood Glucose Monitoring; 6MWT: Six Minute Walk test; DB: Deep Breathing Exercise; EKG: Electrocardiogram; BNP: Brain Naturistic Peptide
